# SIDE—A Unified Framework for Simultaneously Dehazing and Enhancement of Nighttime Hazy Images

**DOI:** 10.3390/s20185300

**Published:** 2020-09-16

**Authors:** Renjie He, Xintao Guo, Zhongke Shi

**Affiliations:** 1 School of Automation, Northwestern Polytechnical University, Xi’an 710129, China; zkeshi@nwpu.edu.cn; 2 School of Electronics and Information, Northwestern Polytechnical University, Xi’an 710129, China; guoxintao@mail.nwpu.edu.cn

**Keywords:** nighttime dehazing, halo removal, Retinex, image enhancement

## Abstract

Single image dehazing is a difficult problem because of its ill-posed nature. Increasing attention has been paid recently as its high potential applications in many visual tasks. Although single image dehazing has made remarkable progress in recent years, they are mainly designed for haze removal in daytime. In nighttime, dehazing is more challenging where most daytime dehazing methods become invalid due to multiple scattering phenomena, and non-uniformly distributed dim ambient illumination. While a few approaches have been proposed for nighttime image dehazing, low ambient light is actually ignored. In this paper, we propose a novel unified nighttime hazy image enhancement framework to address the problems of both haze removal and illumination enhancement simultaneously. Specifically, both halo artifacts caused by multiple scattering and non-uniformly distributed ambient illumination existing in low-light hazy conditions are considered for the first time in our approach. More importantly, most current daytime dehazing methods can be effectively incorporated into nighttime dehazing task based on our framework. Firstly, we decompose the observed hazy image into a halo layer and a scene layer to remove the influence of multiple scattering. After that, we estimate the spatially varying ambient illumination based on the Retinex theory. We then employ the classic daytime dehazing methods to recover the scene radiance. Finally, we generate the dehazing result by combining the adjusted ambient illumination and the scene radiance. Compared with various daytime dehazing methods and the state-of-the-art nighttime dehazing methods, both quantitative and qualitative experimental results on both real-world and synthetic hazy image datasets demonstrate the superiority of our framework in terms of halo mitigation, visibility improvement and color preservation.

## 1. Introduction

Images captured in outdoor scene are often degraded by interaction of atmospheric phenomena. The phenomena such as haze, fog and smoke are mainly generated by the substantial presence of suspended atmospheric particles which absorb, emit or scatter light. As a result, acquired outdoor scenery images are often of low visual quality, such as reduced contrast, limited visibility, and weak color fidelity. The performance of computer vision-based algorithms like detection, recognition, and surveillance are severely limited under such hazy conditions. The goal of image dehazing is to mitigate the influence of haze and recover a clear scene image, which is beneficial for both computational photography and computer vision applications. Based on analysis of the mechanism of haze formation [1], a number of approaches have been proposed in the past decades to solve this challenging problem relying on various image assumptions and priors [2,3,4,5,6,7,8]. While most existing methods are focused on daytime image dehazing, nighttime image dehazing remains a challenging problem. During the night, the performances of existing dehazing methods are significantly limited, because the priors and assumptions of these methods become invalid due to the weak illumination and multiple scattering of the scene ambient light.

Unlike uniformly distributed sunlight as the dominate light source in daytime, illumination varies spatially during the night. In addition, the overall brightness of the scene could be extremely weak, resulting failures of currently used priors and assumptions, such as the dark channel prior [2] and the color attenuation prior [6]. Moreover, significant halo effect caused by multiple scattering can be observed around light sources in hazy night, which is not considered in the commonly used daytime dehazing model. In order to overcome these problems, several techniques have been introduced with novel assumptions recent years, such as bright alpha blending [9], color transfer [10], maximum reflection prior [11] illumination correction [12], glow removal [13] and image fusion [14,15]. However, although the nighttime haze can be removed from the observed images, color distortion still exist in the dehazed results. In addition, visibility and scene details are reduced and hidden due to the low ambient illumination. While various methods have been proposed for low-light image enhancement and have achieved satisfying results [16,17,18,19], practically, dehazing and brightening are regarded as two independent problems in nighttime image processing. As the result, ambient illumination remains dim in dehazed nighttime images, while haze remains in the low-light enhancement results. Although the result can be obtained by a dehazing-and-enhancing flow, severe color distortion could appear. Therefore, it is argued that both brightness enhancement and dehazing are crucial for visibility improvement of nighttime hazy images.

In this paper, a novel unified framework for SImultaneously Dehazing and Enhancement of nighttime hazy images (SIDE) is proposed by considering both halo mitigation and ambient illumination enhancement. More importantly, the classic daytime dehazing methods can be effectively incorporated into nighttime dehazing based on the proposed framework. Specifically, a layer extraction approach is firstly introduced to mitigate the halo artifacts caused by multiple scattering. Then, a Retinex based decomposition is proposed to estimate the spatially varying ambient illumination. After that, daytime dehazing methods can be applied to recover the scene radiance. To the best of our knowledge, the proposed SIDE is the first attempt which considers both haze removal and illumination enhancement for nighttime hazy images. Experimental results on both real-world and synthetic datasets demonstrate the effectiveness of the proposed framework for classic daytime dehazing methods under nighttime hazy conditions. In the comparisons with the state-of-the-art nighttime dehazing methods, both the quantitative and qualitative evaluations indicate the superiority of our proposed SIDE in terms of halo mitigation, visibility improvement and color preservation.

The rest of the paper is organized as follows—Section 2 introduces related work and limitations in existing image dehazing and low-light enhancement. Section 3 throughly describes the proposed SIDE. Section 4 shows the experimental results and analysis. Finally, Section 5 concludes the work.

## 2. Related Work

In hazy days, a beam of light traveling through the atmosphere is attenuated along the incident path and is scattered to other directions. Sensors not only receive the attenuated reflection from the scene objects, but also the additive light in the atmosphere. To describe the formation of the hazy imaging process, in computer vision, a hazy image can be mathematically modeled as the pixel-wise convex combination of the scene radiance and the airlight according to the Koschmieder’s law as follows:

(1) $$I\left(x\right)=J\left(x\right)t\left(x\right)+A\left(1-t\left(x\right)\right),$$

where $I\left(x\right)$ and $J\left(x\right)$ represent the observed hazy image and the haze-free scene radiance in RGB color space, respectively. A is the global atmospheric light, which is assumed to be spatially constant. x indicates the pixel index. $t\left(x\right)\in \left[0,1\right]$ is the depth dependent medium transmission function describing the portion of the light that reaches the camera without scattering, which can be expressed as follows:

(2) $$t\left(x\right)=e^{-\beta d(x)},$$

where β is the scattering coefficient of the atmosphere medium and *d* is the scene depth.

Based on single scattering haze model in (1), various approaches have been proposed to address the single image dehazing problem [1,2,3,4,20,21,22,23]. In recent years, encouraged by the milestone progress in single image dehazing—known as the Dark Channel Prior [2]—the performance of single image dehazing has been improved continuously [4,5,6,7,24,25,26,27]. With the development of machine learning technique, learning-based methods, especially the convolutional neural network (CNN) based methods, have also been introduced to single image dehazing [28,29,30,31,32,33,34,35,36]. Cai et al. proposed an end-to-end DehazeNet to estimate transmission map [29]. The scene radiance was then recovered according to the single scattering model. Ren et al. further improved the accuracy of transmission estimation by introducing a multi-scale deep neural network [30]. Li et al. introduced a lightweight end-to-end CNN based AOD-Net for image dehazing by generating the haze-free images directly [31]. Zhang et al. also addressed the end-to-end dehazing problem under a deep learning based densely connected pyramid dehazing network [34]. Approaches using generative adversarial networks have also been studied in recent years [37,38,39,40] Although these methods are capable of recovering satisfying results in daytime, their performances on nighttime dehazing are quite limited. In addition, learning based methods, especially the data-driven deep learning based approaches rely on the sufficient training data. However, datasets containing a large number of paired earl nighttime hazy images and corresponding clear scene images are practically unable to obtain due to the complex environmental illumination.

Compared with daytime dehazing, less attention has been paid to nighttime dehazing. Pei and Lee [10] proposed a color transfer method for nighttime hazy image mapping, haze was removed using a modified dark channel prior. Although the visibility could be increased, their results are visually unrealistic. Zhang et al. introduced an imaging model that combines gamma correction and color correction [12]. Although their results are visually better, a severe halo effect is also observed. They further proposed a maximum reflectance prior to estimate ambient illumination [11]. However, their method has limitation in illuminant regions. Li et al. addressed the halo effect in nighttime dehazing by introducing an atmospheric point spread function [13]. They removed the glow around light sources through layer decomposition. However, the illumination in the dehazed results remain dim. Ancuti et al. investigated the local airlight estimation and introduced a multi-scale fusion technique for both daytime and nighttime hazy image enhancement by employing a patch-based dark channel prior [14,15].

However, the patch size requires carefully selection. More recently, Yu et al. proposed a pixel-wise alpha blending method to improve the dark channel prior, and the illumination is estimated using guided filter [9]. Although the color constancy is well preserved, halo effect is still obvious due to the use of guided filter. Lou et al. constructed a linear model to connect transmission and haze-relevant features and employed a learning approach to solve the model [41]. Kuanar et al. introduced a CNN based DeGlow model with a embedded DeHaze module for nighttime dehazing [42].

The main limitation of the single scattering model is that Equation (1) only considers the single scattering effect, which means each pixel in the obtained image I(x) corresponds to a sole scene pixel in J(x), while the flux scattered in the other directions by each particle is ignored. The value of a pixel in the observed image is not only composed by the direct attenuation and airlight, but also influenced by its neighbor points due to multiple scattering phenomenon. In daytime, constant atmospheric light is presumed since the homogeneous sunlight is the main light source in the scene. However, the ambient illumination is no longer uniformly distributed during the night. In addition, the inhomogeneous ambient light generated by multicolor light sources varies spatially, and the influence of multiple scattering becomes significant.

On the other hand, continues contributions have been introduced for low-light image enhancement. Based on the Retinex theory, early methods enhance the low-light images by removing illumination component with Gaussian filtering [43,44,45]. However, due to the ill-posed nature of the Retinex model, results are often over-enhanced unnaturally. To effectively overcome such a problem, many recent works resort to variational methods by applying priors and assumptions on the illumination and reflectance with different regularized models [46,47,48,49,50]. Kimmel et al. introduced a smooth illumination prior in the regularization term [46]. Ng et al. proposed a $\ell_{2}$ fidelity prior with TV based regularized model by considering both the illumination and the reflectance [47]. Inspired by Ng’s work, Wang et al. proposed a constrained variational model with barrier functionals [50]. Based on the assumption that the illumination is spatial smooth and the reflectance is piece-wise continuous, Fu et al. proposed a probabilistic algorithm and a weighted variational method to decompose the illumination and the reflectance simultaneously [51,52]. Guo et al. proposed a $\ell_{1}$ norm based regularization framework to refine an initial illumination map under a structure-aware prior [17]. Learning based methods have also been developed for low-light image enhancement [53,54,55,56,57,58]. Although visibility and contrast could be increased, these approaches can not handle haze in the scene.

In this paper, we address both haze removal and illumination enhancement for nighttime hazy images in a unified framework. In order to better understand and describe nighttime haze, halo effect and spatially varying illumination should be considered. Inspired by Li’s work [13], we employ following nighttime haze model in our work:

(3) $$I\left(x\right)=J\left(x\right)t\left(x\right)+L\left(x\right)\left(1-t(x)\right)+H\left(x\right),$$

where L(x) denotes the varying ambient illumination, and H(x) indicates the additive halo layer.

## 3. Methodology of the Proposed SIDE

**Figure 1:**
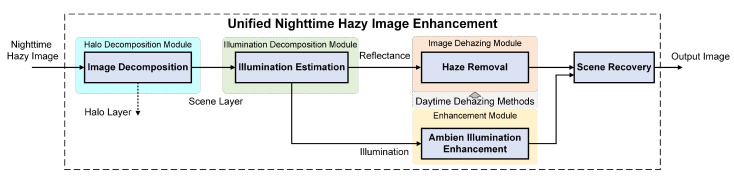
Framework of the proposed SImultaneously Dehazing and Enhancement of nighttime hazy images (SIDE).

The scheme of the proposed SIDE is illustrated in Figure 1, which includes Halo Decomposition Module, Illumination Decomposition Module, Image Dehazing Module and Enhancement Module. We firstly introduce a halo extraction module to mitigate the halo artifacts caused by multiple scattering After that, we propose a Retinex based illumination decomposition method to estimate the spatially varying ambient illumination. With the illumination extracted, the classic daytime dehazing methods are employed for haze removal. Finally, we generate the output scene image by combining the adjusted illumination and dehazed scene layer. We will express each module in detail in the following subsections.

### 3.1. Halo Decomposition Module

As discussed above, one major problem of nighttime hazy images is detail and visibility degradation of objects around light sources caused by the multiple scattering of nearby illuminants. Inspired by Li’s work [13], an observed hazy image I(x) can be modeled as a linear superimposition of a scene layer S which contains haze and scene information, and a halo layer H which indicates halos and glows around illuminants. Consequentially, Equation (3) can thus be written as:

(4) I(x)=S(x)+H(x),

where S and H indicate the scene layer and the halo layer, respectively.

The halo layer has the characteristics of high intensities and smooth variation around light sources, wheres hazy scene layer itself only contains scene structure and texture details with relatively dim brightness. It is noticed that the gradients of halo patches has a sharper and sparser distribution compared with the non-halo patches. Therefore, a probabilistic model with prior knowledge on gradient distribution of the two layers can be employed to extract the halo layer. By assuming S and H are independent, the optimized S and H can be decomposed by minimizing the object function as follows:

(5) $$\min_{S,H}\sum_{x}\left\{\sum_{\partial_{i}\in \Omega_{S}}\left|\partial_{i}*S\left(x\right)\right|+\sum_{\partial_{j}\in \Omega_{H}}\frac{\lambda }{2}{∥\partial_{j}*H\left(x\right)∥}^{2}\right\},$$

where ∗ denotes the convolution operator. *∂* indicates the derivative filters in sets $\Omega_{S}=\left\{\left[1,-1\right],{\left[1,-1\right]}^{T}\right\}$ and $\Omega_{H}=\left\{\left[1,-2,1\right],{\left[1,-2,1\right]}^{T}\right\}$, which contain the first order derivative filters in two directions, and the second order Laplacian filter, respectively. The scale weight λ controls the smoothness of the halo image layer.

According to convolutional system theory, the convolutional result of a signal and a finite-length sequence can be expressed with the product of a Toeplitz matrix and the signal, where the Toeplitz matrix is uniquely determined by the finite-length sequence. By substituting H=I−S, Equation (5) can be rewritten as follows:

(6) $$\min_{S}\sum_{x}\left\{\sum_{i\in \Omega_{S}}\left|D_{i}S\left(x\right)\right|+\sum_{j\in \Omega_{H}}\frac{\lambda }{2}{∥D_{j}I\left(x\right)-D_{j}S\left(x\right)∥}^{2}\right\},$$

where $D_{k}S\left(x\right)$ indicates the elements of the Toeplitz matrix generated by the convolutional kernel $\partial_{k}$.

**Figure 2:**
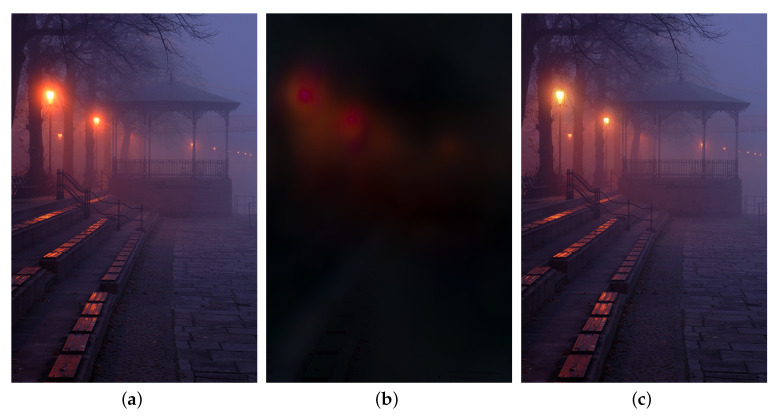
Illustration of halo layer extraction. (**a**): a nighttime hazy image, (**b**): the halo layer, (**c**): the scene layer.

The non-convex problem in (6) can be optimized via the alternating direction method of multipliers (ADMM) as in References [59,60]. Figure 2 shows a result of halo layer separation. It is observed in the scene layer that halo effect around light sources is mitigated, compared with the observed nighttime hazy image.

### 3.2. Illumination Decomposition Module

With the halo layer H extracted, the dehazing problem becomes:

(7) $$S\left(x\right)=J\left(x\right)t\left(x\right)+L\left(x\right)\left(1-t(x)\right).$$

Unlike the commonly used haze imaging model in (1) which assumes a global constant atmospheric light, the ambient illumination in our work is assumed to be an inhomogeneous and spatially-varying map L(x). Consequentially, the local maximum assumption of atmospheric light in daytime dehazing no longer holds for estimating L(x) during nighttime. Inspired by the Retinex theory which assumes an observed image as the combination of reflectance and illumination, we resort to the illumination decomposition model to overcome these limitations.

According to the Retinex theory [61], the scene layer image can be formulated as the pixel-wise product of a reflectance component and a light-dependent illumination component as follows:

(8) S=R(x)∘L(x),

where R is the reflectance component, and L is the illumination component. ∘ represents the pixel-wise multiplication.

Denoting J(x)=L(x)ρ(x), Equation (7) can be reformulated in a Retinex-like pattern as follows:

(9) S(x)=L(x)[ρ(x)t(x)+1−t(x)],

where ρ is the intrinsic reflectance of objects in the scene [20,62].

The illumination is presumed to be spatially smooth and contains the overall structure, which shares the same characteristic of ambient illumination of the scene layer, therefore, (9) can be solved by minimizing the following objective function:

(10) $$\min_{R,L,\omega }{∥R\circ L-S∥}_{F}^{2}+{\mathrm{TGV}}_{\alpha }^{2}\left(L\right)+\beta {∥\nabla R-\nabla S∥}_{0},$$

where R=ρ(x)t(x)+1−t(x), β is the regularization parameter and ∇ is the first order differential operator. ${\mathrm{TGV}}_{\alpha }^{2}\left(L\right)$ indicates the second order TGV with respect to L, which can be expressed in terms of the following $\ell_{1}$ minimization problem:

(11) $${\mathrm{TGV}}_{\alpha }^{2}\left(L\right)=\min_{\omega }\;\alpha_{1}{∥D\left(\nabla L-\omega \right)∥}_{1}+\alpha_{0}{∥\nabla \omega ∥}_{1},$$

where $\alpha \in \{\alpha_{0},\alpha_{1}\}$ is a weighting vector and ω is a vector field with low variation. D is a diffusion tensor formulated as follows:

(12) $$D=\exp\left(-{\zeta |\nabla S|}^{\mu }\right)nn^{T}+n^{⊥}n^{⊥T},$$

where $n=\nabla S/\left|\nabla S\right|$ indicates the normalized direction of the image gradient, and $n^{⊥}$ is the normal vector to the gradient. ζ and μ are parameters controlling the magnitude and the sharpness of D.

**Figure 3:**
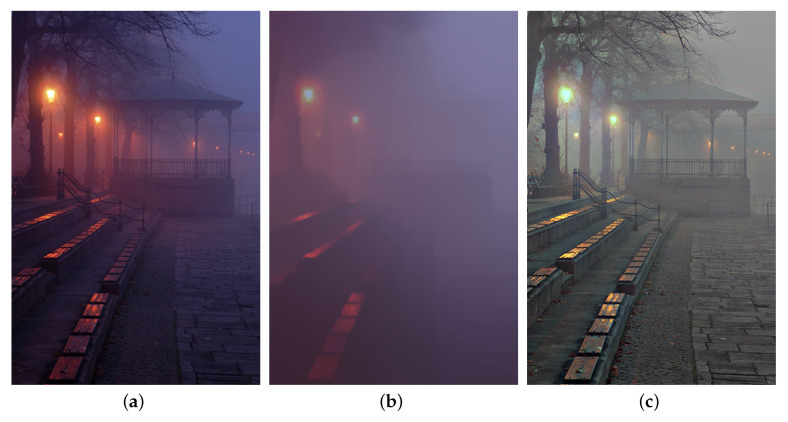
A sample of ambient illumination decomposition. (**a**): scene layer, (**b**): estimated ambient illumination, (**c**): estimated reflectance.

In practice, we apply the $\ell_{1}$ norm as the convex relaxation [63] of the original $\ell_{0}$ minimization problem. In addition, (10) is a non-convex and non-smooth optimization problem because of the adoption of pixel-wise multiplication and TGV regularization. An alternating direction method of multipliers (ADMM) [59,60] is adopted to solve it. Figure 3 demonstrates an example of ambient illumination estimation. It is observed the unevenly distributed ambient illumination is effectively estimated. Color distortion caused by artificial illuminant is also corrected in the reflectance.

### 3.3. Scene Recovery Module

As expressed in (9), the reflectance component R in Equation (8) is composed as follows:

(13) R(x)=ρ(x)t(x)+1−t(x),

which can be expressed in the following formation:

(14) $$R\left(x\right)=J_{1}\left(x\right)t\left(x\right)+A_{1}\left(1-t\left(x\right)\right).$$

It can be easily observed that Equation (13) has the similar formation of the original single scattering model in Equation (1) with the atmospheric light as $A_{1}={\left(1,1,1\right)}^{T}$ and the scene radiance $J_{1}\left(x\right)=\rho \left(x\right)$. Therefore, various assumptions and priors of existing daytime dehazing approaches can be employed to effectively solve Equation (13). In addition, to preserve the naturalness of the scene, we also manipulate the ambient illumination L(x) with gamma transformation to increase the global brightness. We will demonstrate and analyze the results in the next section.

## 4. Experimental Results and Analysis

In this section, we firstly demonstrate the effectiveness of the proposed SIDE in terms of halo layer extraction. After that, we compare the performances of various daytime dehazing methods with and without the proposed SIDE. In addition, comparisons with conventional low-light image enhancement methods are also illustrated. Next, comprehensive experiments are conducted to demonstrate the superiority of the proposed SIDE. Since it is hard to obtain the nighttime haze and daytime haze-free image pairs, the proposed SIDE is evaluated on Zhang’s datasets [11], which contains 20 real world nighttime hazy images. We also test the proposed SIDE on synthesized nighttime hazy images for quantitative comparisons. The proposed algorithm is implemented using MATLAB 2019b on PC with 9700K CPU and 32GB RAM. In the implementation, parameter λ is set to 3 for halo layer extraction, the regularization parameters for ambient illumination decomposition are set as $\alpha_{0}=0.5$, $\alpha_{0}=0.05$. While most conventional daytime dehazing methods can be applied for scene radiance recovery, He’s dark channel prior (DCP) [2], Meng’s boundary constraint method (BC) [4], Color Attenuation Prior (CAP) [6], and Berman’s non-local method (NL) [7] are selected for demonstrations. The performance of the proposed SIDE is also compared with several state-of-the-art nighttime dehazing approaches, including Zhang’s Maximum Reflectance Prior (MRP) [11,12], Li’s Glow and Multiple Light Colors (GMLC) [13], Yu’s Pixel-wise Alpha Blending (PAB) [9] and Lou’s Haze Density Features (HDF) [41], where the parameters are set as defined in the references.

### 4.1. Results on Hazy Scene Estimation

**Figure 4:**
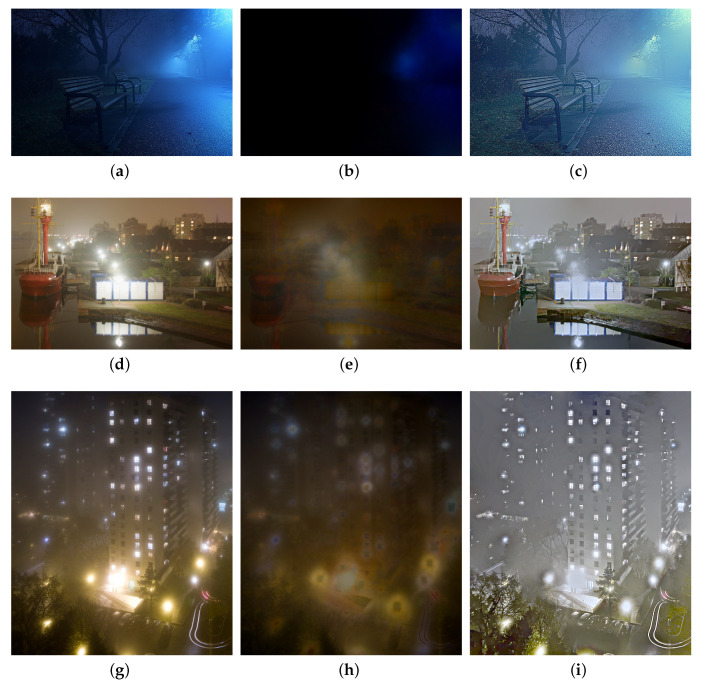
Illustration of halo layer extraction. (**a**,**d**,**g**): nighttime hazy images, (**b**,**e**,**h**): the halo layers, (**c**,**f**,**i**): the scene layers.

To demonstrate the effectiveness of our halo layer extraction, we first illustrate the results of the extracted halo layer and scene layer, in Figure 4. It can be seen that the halos around illuminants are effectively mitigated in the scene layers.

### 4.2. Verification on Daytime Dehazing Methods

**Figure 5:**
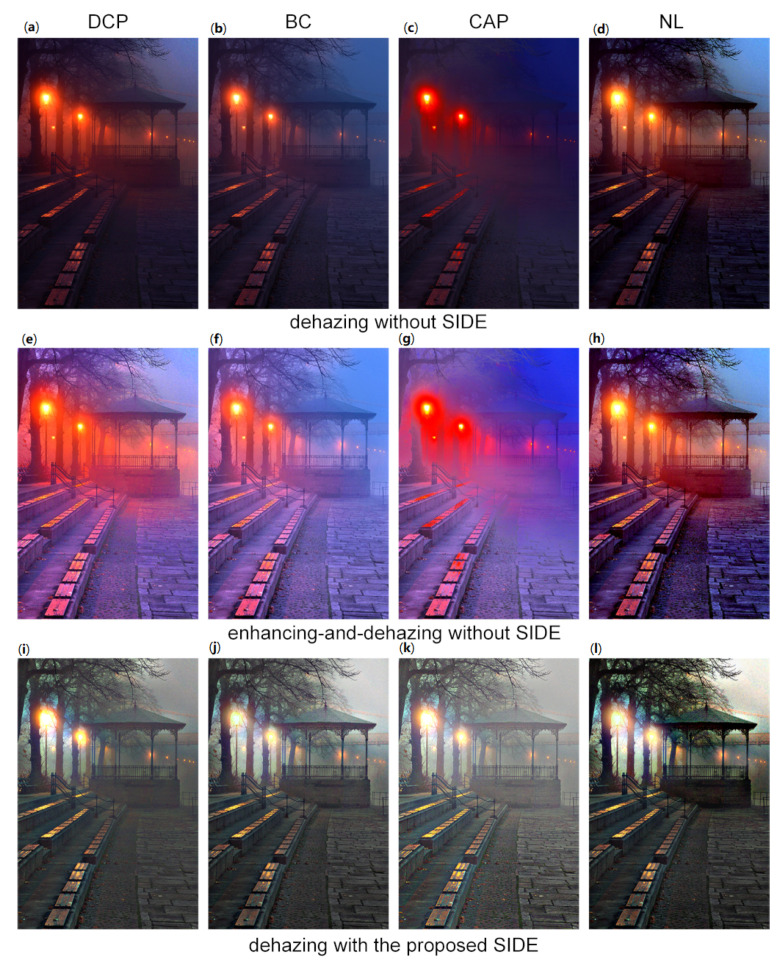
Illustration of the proposed SIDE. (**a**–**d**): results of conventional daytime dehazing methods without SIDE; (**e**–**h**): results with the enhancing-and-dehazing flow (enhanced by LIME [17]); (**i**–**l**): results of corresponding methods with the proposed SIDE. From left to right: dark channel prior (DCP) [2], boundary constraint method (BC) [4], Color Attenuation Prior (CAP) [6] and Berman’s non-local method (NL) [7].

To demonstrate the effectiveness of the proposed SIDE, we compare the performances of five classic daytime dehazing methods with and without SIDE on test nighttime hazy images. As shown in Figure 5a–d show the dehazing results without our SIDE using He’s dark channel prior (DCP) [2], Meng’s boundary constraint method (BC) [4], Color Attenuation Prior (CAP) [6], and Berman’s non-local method (NL) [7], respectively. Figure 5e–h show the results in the enhancing-and-dehazing flow of the corresponding methods, while the observed hazy image is firstly enhanced using LIME [17]. The bottom row of Figure 5 show the dehazing results of corresponding methods with the proposed SIDE. It is observed in the top row that applying daytime dehazing methods directly on nighttime hazy images would cause halo artifacts, color distortion, contrast reduction in outputs. Details are also lost in dark regions in the results. In the middle row, halo artifacts and color distortion become more serious after enhancement by LIME [17]. It is also noticed in Figure 5g that the visibility is worse after the enhancement. On the contrary, the results in the bottom row have better visibility and ambient color. In addition, the halo artifacts are significantly mitigate. It is clearly that these daytime dehazing methods can be effectively applied to nighttime dehazing under the proposed SIDE framework.

**Figure 6:**
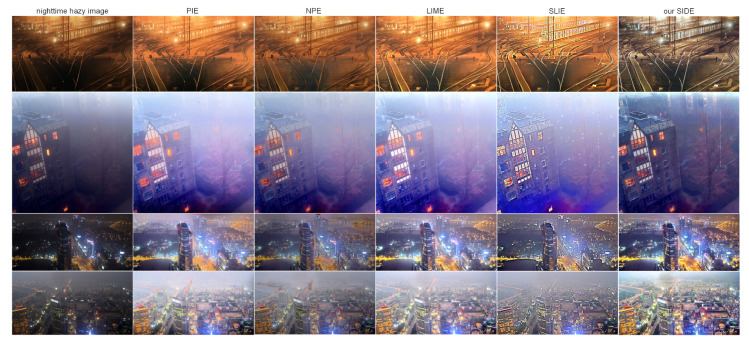
Comparisons of conventional low-light enhancement methods. From left to right: real nighttime hazy images, results of low-light enhancement methods PIE [51], NPE [49], LIME [17], SLIE [18] and the result of proposed SIDE.

We also compare our SIDE with different low-light enhancement methods in Figure 6. Specifically, the probabilistic image enhancement method (PIE) [51], the naturalness preserved enhancement method (NPE) [49], the low-light image enhancement via illumination map estimation (LIME) [17] and the structure-revealing low-light image enhancement method (SLIE) [18] are employed for comparisons. It is easily observed that although existing low-light enhancement methods can significantly increase the contrast of the nighttime hazy images, haze remains in the enhanced results.

**Figure 7:**
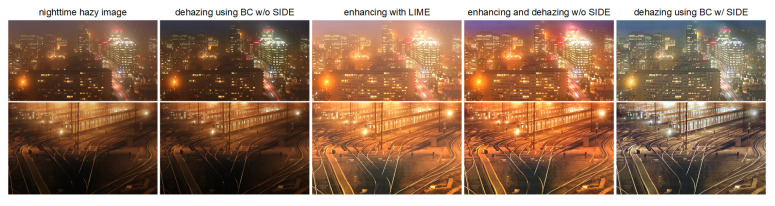
Comparisons of conventional dehazing methods and low-light enhancement methods. From left to right: observed nighttime hazy images, dehazing results using BC [4] directly, enhancement results using LIME [17], dehazing results after enhancement, dehazing results using BC with the proposed SIDE.

To further verify the effectiveness of the proposed SIDE, we compare the results of our SIDE with the results using traditional daytime dehazing method BC [4] directly, using the low-light enhancement method LIME [17], and using the enhancing and dehazing flow, respectively. As shown in Figure 7, although conventional daytime dehazing methods can mitigate haze in certain regions, residual haze can be observed globally. In addition, the globally illumination remains dark in the dehazing results. On the other hand, while traditional low-light enhancement methods are capable of increasing visibility, haze and halo artifacts become severer. It is also noticed that the performances of dehazing-after-enhancement are not satisfying. Clearly, based on the proposed SIDE, the conventional daytime dehazing methods can be employed in nighttime dehazing.

### 4.3. Qualitative Comparisons on Real Nighttime Hazy Images

**Figure 8:**
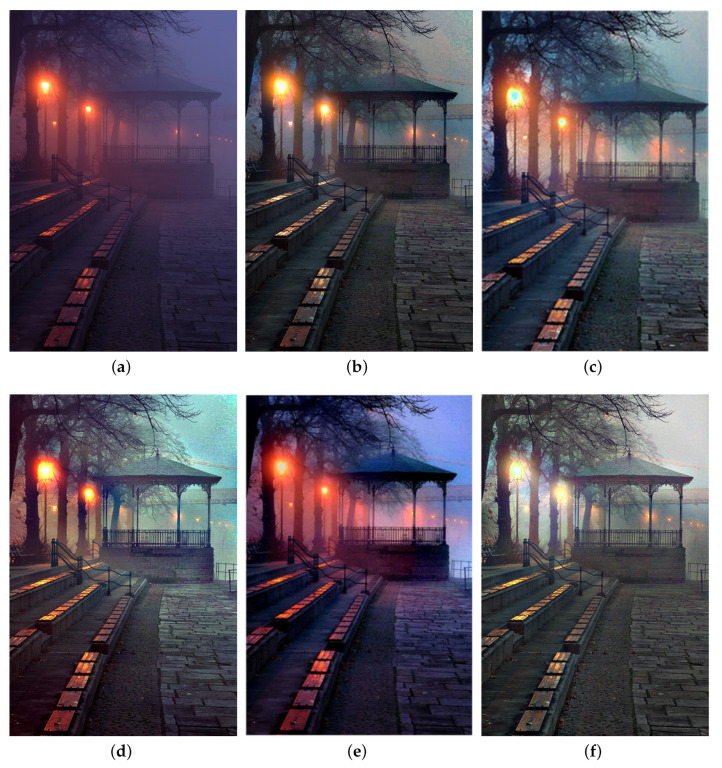
Comparisons with other nighttime dehazing methods on test image *Pavilion*. (**a**): the nighttime hazy image, (**b**): Zhang’s Maximum Reflectance Prior (MRP) [11] result, (**c**): Lou’s Haze Density Features (HDF) [41] result, (**d**): Li’s Glow and Multiple Light Colors (GMLC) [13] result, (**e**): Yu’s Pixel-wise Alpha Blending (PAB) [9] result, (**f**): SIDE result.

**Figure 9:**
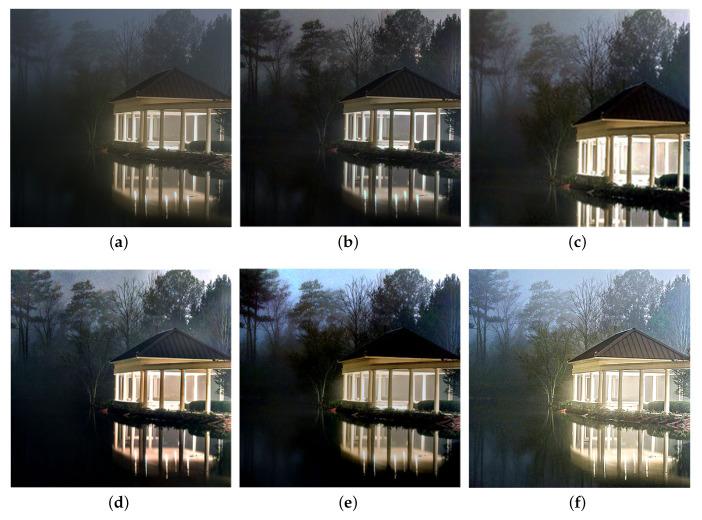
Comparisons with other nighttime dehazing methods on test image *Lake*. (**a**): the nighttime hazy image, (**b**): MRP [11] result, (**c**): HDF [41] result, (**d**): GMLC [13] result, (**e**): PAB [9] result, (**f**): SIDE result.

**Figure 10:**
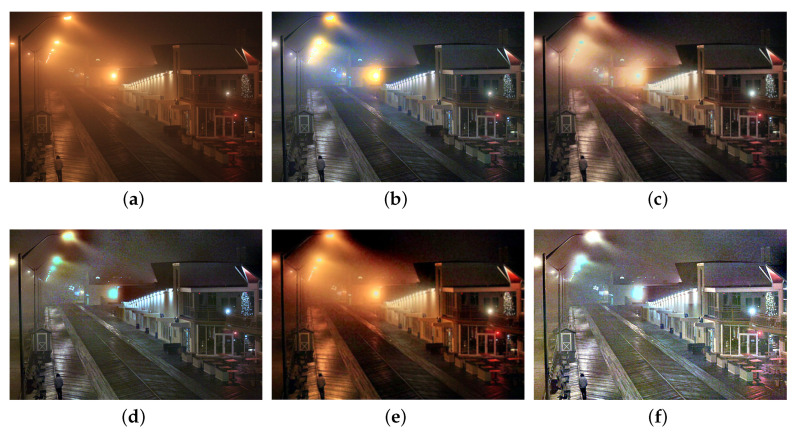
Comparisons with other nighttime dehazing methods on test image *Street*. (**a**): the nighttime hazy image, (**b**): MRP [11] result, (**c**): HDF [41] result, (**d**): GMLC [13] result, (**e**): PAB [9] result, (**f**): SIDE result.

**Figure 11:**
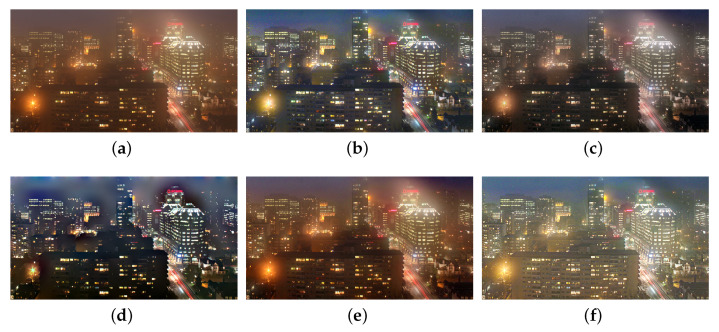
Comparisons with other nighttime dehazing methods on test image *Cityscape*. (**a**): the nighttime hazy image, (**b**): MRP [11] result, (**c**): HDF [41] result, (**d**): GMLC [13] result, (**e**): PAB [9] result, (**f**): SIDE result.

**Figure 12:**
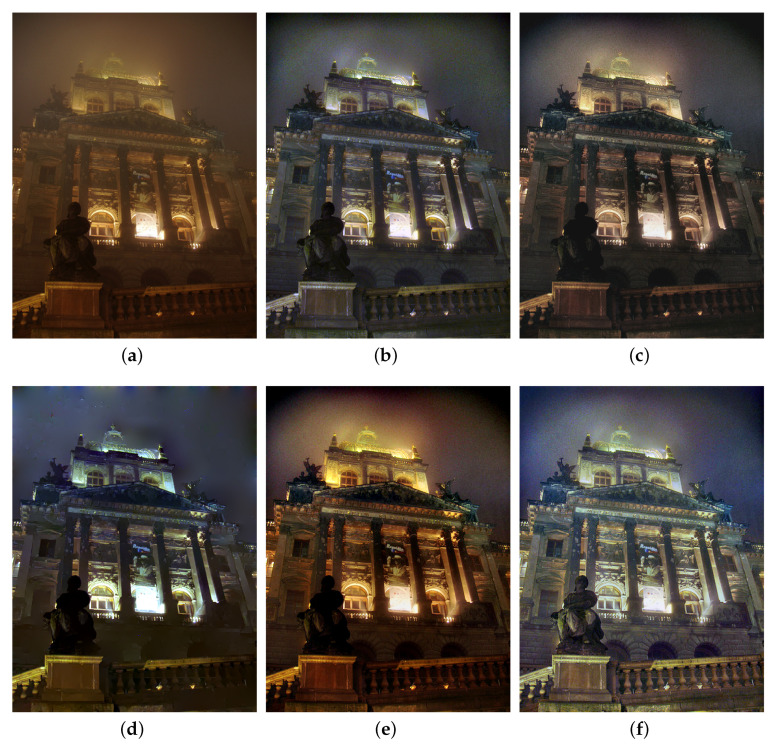
Comparisons with other nighttime dehazing methods on test image *Church*. (**a**): the nighttime hazy image, (**b**): MRP [11] result, (**c**): HDF [41] result, (**d**): GMLC [13] result, (**e**): PAB [9] result, (**f**): SIDE result.

**Figure 13:**
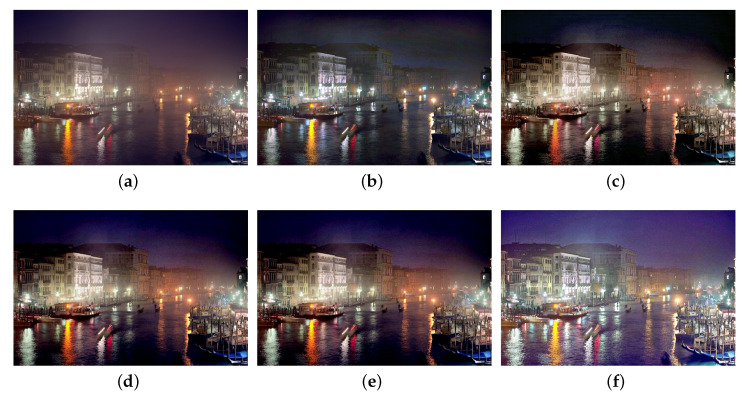
Comparisons with other nighttime dehazing methods on test image *Riverside*. (**a**): the nighttime hazy image, (**b**): MRP [11] result, (**c**): HDF [41] result, (**d**): GMLC [13] result, (**e**): PAB [9] result, (**f**): SIDE result.

**Figure 14:**
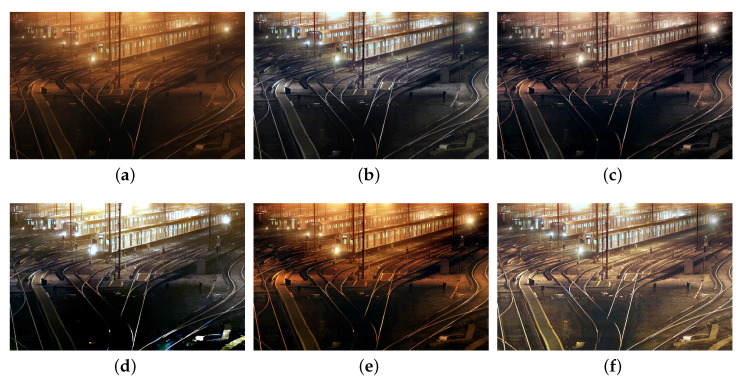
Comparisons with other nighttime dehazing methods on test image *Railway*. (**a**): the nighttime hazy image, (**b**): MRP [11] result, (**c**): HDF [41] result, (**d**): GMLC [13] result, (**e**): PAB [9] result, (**f**): SIDE result.

We also compare the proposed SIDE with existing nighttime dehazing methods on real nighttime hazy images. In our implementation, Meng’s BC [4] is employed for scene recovery. Figure 8, Figure 9, Figure 10, Figure 11, Figure 12, Figure 13 and Figure 14 show seven comparison results on real test images.

**Figure 15:**
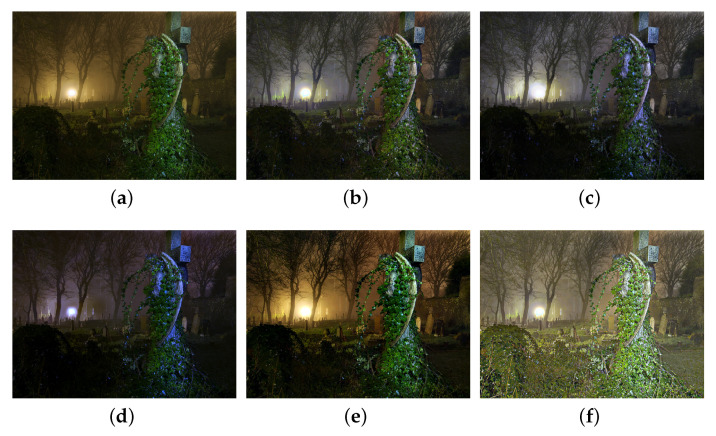
Comparisons with other nighttime dehazing methods on test image *Tomb*. (**a**): the nighttime hazy image, (**b**): MRP [11] result, (**c**): HDF [41] result, (**d**): GMLC [13] result, (**e**): PAB [9] result, (**f**): SIDE result.

As observed in Figure 8, all the compared methods are capable of removing haze for nighttime hazy images. Although PAB [9] is capable of increasing the contrast of the scene to a certain degree, apparent halo can be observed in Figure 8e. In addition, the brightness of the result remains dim. While MRP [11], HDF [41], GMLC [13] and the proposed SIDE can significantly increase the visibility and suppress halo effect, the proposed SIDE has better contrast improvement in local regions. Color distortion can be seen in grove regions in MRP [11] and GMLC [13], while it is more natural in our result. HDF [41] also results in residual haze in the grove region. Although no ground-truth reference image is available, the result of SIDE has the best subjective performance on color constancy. In Figure 9, while all the methods are able to remove haze and increase scene visibility, the proposed SIDE has the best performance on detail recovery. Reflection of the woods in the water is recovered well in our result. In Figure 10, it is noticed in MRP [11] result that, over-saturation around lamps can be observed and halos are also significant in the dehazed result. Although haze is removed in PAB [9] and HDF [41] results, halo artifacts still exist. Moreover, the result suffers from dim and distorted illumination. While both GMLC [13] and our SIDE are capable of mitigating halo artifacts significantly, more details and better visibility are observed in our result. In Figure 11, halo artifacts are observed in HDF and PAB results. In GMLC [13] result, although halos around illuminants are well suppressed, structural halos are generated around buildings. Moreover, details in dark regions are lost. As observed in Figure 11f, our result has the best performance in terms of halo suppression and detail recovery. Figure 12 shows the comparison on the real test image *Church*. All the nighttime dehazing methods are capable of removing haze from the scene effectively. Although GMLC [13] can suppress halos around artificial illumination, structural halo are noticed around build boundary. In addition, while all the compared methods have limitation on recovering scene details hiding in the dark, it is easily noticed that our result has the best performance on halo mitigation and detail recovery. Figure 13 demonstrates the comparison on the test image *Riverside*. Since the haze is quite light in the scene, all the methods ca effectively remove the haze and halos around lights are not significant. As observed in the comparison, our method can achieve the best visual performance in terms of detail recovery and globally illumination enhancement. Figure 14 presents a comparison on the test image *Railway*, where the color temperature of the illumination is quite warm, and the illumination is mainly distributed at the top of the image. As observed in the comparison, all the methods can increase the visibility of the hazy image. Halos are noticed in MRP [11] and GMLC [13] results due to over-saturation in bright regions. It is observed in our result that halos are well suppressed and details in foreground regions are significantly enhanced. However, it is also noticed in our result that lights at the top are slightly over-enhanced due to the illumination enhancement. Figure 15 presents a comparison on the test image *Tomb*, where the flashlight was activated. As seen in the comparison, halos are suppressed in results by GMLC and our SIDE. However, all the compared nighttime dehazing methods have limitations when recovering details of foreground regions (left bottom corner). Figure 15 shows a comparison on the test image *Building*, which shows a cityview in a hazy night. As observed in the comparison, GMLC generates obvious halo artifacts around building boundary, and HDF slightly increase the visibility.

**Figure 16:**
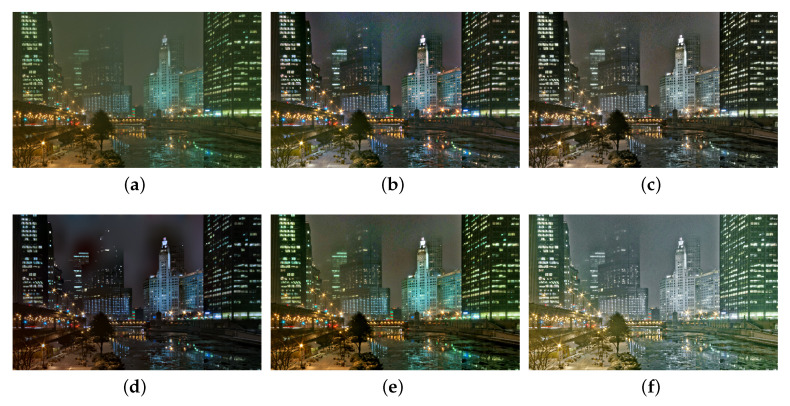
Comparisons with other nighttime dehazing methods on test image *Building*. (**a**): the nighttime hazy image, (**b**): MRP [11] result, (**c**): HDF [41] result, (**d**): GMLC [13] result, (**e**): PAB [9] result, (**f**): SIDE result.

Since no ground-truth images are available, the no-reference haze density metric (HDM) and the no-reference image quality metric for contrast distortion (NIQMC) [64] are employed for evaluation. The HDM includes three evaluators, namely *e*, Σ and $\bar{r}$, which indicate the rate of newly appeared edges, the percentage of pure white or pure black pixels and the mean ratio of edge gradients, respectively. A better dehazed image should have higher values of *e*, $\bar{r}$, NIQMC, and a lower value of Σ, which are indicated by ↓ and ↓, respectively. Table 1 shows the HDM evaluation results of Figure 8, Figure 9, Figure 10, Figure 11, Figure 12, Figure 13, Figure 14, Figure 15 and Figure 16 and the average evaluation result on the real-world dataset by different methods. It is shown that the proposed SIDE achieved the best performance for most indicators. Due to the illumination enhancement of the proposed SIDE, it is also noticed that our method achieves absolute advantage in terms of the metrics $\bar{r}$ and NIQMC.

### 4.4. Comparisons on Synthesized Nighttime Hazy Images

**Figure 17:**
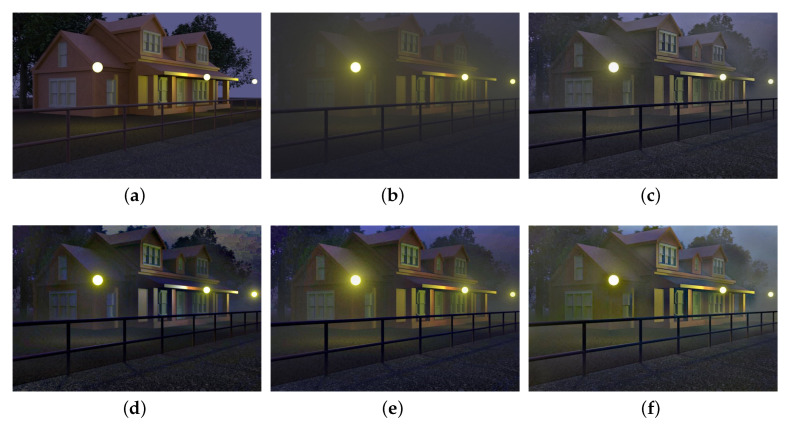
Comparisons on the synthetic test image. (**a**): the ground-truth image, (**b**): result of MRP [11], (**c**): result of HDF [41], (**d**): GMLC [13], (**e**): result of PAB [9], (**f**): result of SIDE.

Besides comparisons on a real image dataset, we also evaluate the performance of the proposed SIDE objectively on synthetic test image according to Li’s work [13], where the hazy image is generated using PBRT. Figure 17 demonstrates the comparison of MRP [11], HDF [41], GMLC [13], PAB [9] and SIDE. Table 2 shows the PSNR and structural similarity (SSIM) evaluation results of different methods. It is observed that our proposed SIDE achieved the best results in both terms of MSE and SSIM, comparing with the existing state-of-the-art nighttime dehazing methods.

## 5. Conclusions

In this paper, we propose a novel unified framework, namely SIDE, to simultaneously remove haze and enhance illumination for nighttime hazy images. Specifically, both halo artifacts caused by multiple scattering and non-uniformly distributed ambient illumination are considered in our approach. In addition, we prove that the conventional daytime dehazing approaches can be effectively incorporated into nighttime dehazing task based on the proposed SIDE. In order to mitigate the halo artifacts caused by multiple scattering, a robust layer decomposition method is firstly introduced to separate the halo layer from the hazy image. A Retinex based illumination decomposition method is then proposed to estimate the non-uniformly distributed ambient illumination. By removing the ambient illumination, the original nighttime dehazing problem can be effectively solved using various daytime dehazing methods. Experimental results demonstrate the effectiveness of the proposed framework for classic daytime dehazing methods under nighttime hazy conditions. In addition, compared with the state-of-the-art nighttime dehazing methods, both quantitative and qualitative comparisons indicate the superiority of the proposed SIDE in terms of halo mitigation, visibility improvement and color preservation.

## Figures and Tables

**Table 1:** Quantitative Comparison on Real-world Benchmark Dataset. The bold indicates the best scores of the quantitative comparisons.

		MRP [11]	HDF [41]	GMLC [13]	PAB [9]	SIDE
*Pavilion*	*e*↑	0.08	0.13	0.15	0.09	**0.17**
	Σ↓	0.01	0.01	**0**	0.03	**0**
	$\bar{r}$↑	4.35	2.97	5.01	4.67	**5.08**
	NIQMC↑	4.67	4.82	4.93	5.01	**5.16**
*Lake*	*e*↑	0.03	0.08	0.13	0.19	**0.23**
	Σ↓	0.01	0.02	**0**	**0**	**0**
	$\bar{r}$↑	2.33	3.54	5.28	5.66	**7.21**
	NIQMC↑	4.91	4.88	5.14	4.99	**5.37**
*Street*	*e*↑	0.20	0.17	0.11	0.09	**0.22**
	Σ↓	0.03	0.18	0.05	0.24	**0.01**
	$\bar{r}$↑	4.55	3.82	4.39	1.61	**4.74**
	NIQMC↑	4.89	4.28	**5.32**	4.47	5.15
*Cityscape*	*e*↑	0.11	0.13	0.02	0.08	**0.19**
	Σ↓	**0.03**	0.44	0.25	0.19	0.05
	$\bar{r}$↑	3.69	3.17	2.01	1.87	**3.96**
	NIQMC↑	3.98	4.96	**5.11**	4.20	5.03
*Church*	*e*↑	0.11	0.10	0.12	0.18	**0.29**
	Σ↓	0.07	0.06	0.08	**0.03**	0.04
	$\bar{r}$↑	4.77	3.83	2.98	3.95	**5.10**
	NIQMC↑	3.97	4.98	5.05	4.19	**5.61**
*Riverside*	*e*↑	0.06	0.09	0.10	0.14	**0.25**
	Σ↓	0.09	0.10	0.04	0.05	**0.03**
	$\bar{r}$↑	2.85	3.68	4.17	4.23	**6.51**
	NIQMC↑	4.71	4.39	4.97	4.85	**5.43**
*Railway*	*e*↑	0.18	0.13	0.15	0.07	**0.27**
	Σ↓	0.08	0.12	0.04	0.16	**0.02**
	$\bar{r}$↑	2.68	2.74	2.30	2.87	**4.98**
	NIQMC↑	4.25	4.19	5.03	5.24	**5.48**
*Tomb*	*e*↑	0.26	0.18	0.09	0.31	**0.45**
	Σ↓	0.03	0.07	0.16	0.02	**0**
	$\bar{r}$↑	5.13	4.25	3.97	5.72	**7.11**
	NIQMC↑	5.04	4.61	4.02	5.26	**6.07**
*Building*	*e*↑	0.11	0.18	0.09	0.21	**0.30**
	Σ↓	0.04	0.06	0.29	0.03	**0.01**
	$\bar{r}$↑	3.79	4.05	2.88	3.81	**4.74**
	NIQMC↑	4.56	4.80	2.73	5.76	**6.20**
Average	*e*↑	0.12	0.16	0.11	0.05	**0.29**
	Σ↓	0.09	0.13	0.08	0.19	**0.04**
	$\bar{r}$↑	3.29	3.45	2.16	2.37	**5.62**
	NIQMC↑	4.80	4.93	5.15	5.04	**5.86**

**Table 2:** Quantitative comparison on the synthetic test image. The bold indicates the best scores of the quantitative comparisons.

	MRP [11]	HDF [41]	GMLC [13]	PAB [9]	SIDE
SSIM	0.7133	0.7558	0.7605	0.7591	**0.7616**
PSNR	17.4122	17.9537	17.2907	17.8926	**18.0025**
